# Vector similarity measures of hesitant fuzzy linguistic term sets and their applications

**DOI:** 10.1371/journal.pone.0189579

**Published:** 2017-12-20

**Authors:** Yongming Song, Jun Hu

**Affiliations:** 1 School of Management and Economics, University of Electronic Science and Technology of China, Chengdu, P. R. China; 2 State-owned Assets Supervision and Administration Commission in Yunnan Province of China, Kunming, P. R. China; Ulm University, GERMANY

## Abstract

In decision making, similarity measure and distance between two objects are crucial to be able to determine the relationship between those objects. Many researchers have received much attention for their research on this subject. In this study, we propose two novel similarity measures between hesitant fuzzy linguistic term sets (HFLTSs). In addition, two extensions of Technique for Order of Preference by Similarity to Ideal Solution (TOPSIS) are proposed in the hesitant fuzzy linguistic environments. Furthermore, an example of an application concerning traditional Chinese medical diagnosis and an MCDM problem have been given to illustrate the applicability and validation of these similarity measures of HFLTSs. Furthermore, the results of examples demonstrate that the Dice and Jaccard similarity measures are more reasonable than the cosine similarity measure with respect to HFLTSs.

## Introduction

Similarity measures and distances are widely used to determine the relationship between two individuals in many domains [1–8], including medical engineering, decision making, pattern recognition, and network comparison. The “classical” similarity measures comprise Dice’s measure, Jaccard’s measure, the cosine formula, the overlap measures, and the correlation coefficient of Pearson [9]. The Dice, Jaccard, and cosine measures are types of vector similarity measures. With the development of fuzzy sets, “classical” similarity measures have been extended to various fuzzy environments [10–24]. Xu et al. [19] introduced the cosine similarity measures for the hesitant fuzzy environment. Zhang et al. [24] defined an integrated similarity measure based on the Dice and cosine measures for intuitionistic fuzzy sets. Ye [22] defined the Dice similarity measure for intuitionistic fuzzy sets. Ye [23] extended the Dice, Jaccard and cosine similarity measures to hesitant fuzzy sets. Through an example, Ye [23] pointed out that the Dice and Jaccard measures are more reasonable than the cosine measure when applied to a hesitant fuzzy set. Chiclana et al. [1] put forward a statistical comparative study of the manner in which five distance functions (Manhattan, Euclidean, cosine, Dice, and Jaccard) affect the consensus process for group decision-making problems.

In some practical problems, because decision makers may exist in a state of hesitation for several linguistic terms with comparison of two methods, such a linguistic term is often insufficient. To deal with this situation, Rodríguez et al. [25] introduced the hesitant fuzzy linguistic term set (HFLTS), which is a strong structure that reflects decision makers’ hesitant attitude [26]. Moreover, different similarity measures for HFLTS have been put forth [15–17], e.g., Liao et al. [16] introduced the cosine similarity measures for HFLTS. In this paper, based on vector similarity measures, we extend Dice and Jaccard measures to HFLTSs and denote them as $D_{HFLTSs}\left(H_{s}^{1},H_{s}^{2}\right)$ and $J_{HFLTSs}\left(H_{s}^{1},H_{s}^{2}\right)$, respectively. Moreover, the $D_{HFLTSs}\left(H_{s}^{1},H_{s}^{2}\right)$- and $J_{HFLTSs}\left(H_{s}^{1},H_{s}^{2}\right)$-distance-based technique for order of preference by similarity to an ideal solution (HFL-TOPSIS) methods are further established. Through examples, it shows that the extended Dice and Jaccard measures with HFLTSs are more reasonable than the cosine measure.

## Similarity measures with hesitant fuzzy linguistic term set

### Vector similarity functions

In the following, we introduce two classical vector similarity measures: Dice similarity [27] and Jaccard similarity [28]. Assuming two vectors, *X* = (*x*_1_,*x*_2_,⋯,*x*_*n*_) and *Y* = (*y*_1_,y_2_,⋯,y_*n*_), we can obtain

$$D\left(X,Y\right)=\frac{2X•Y}{{\left\|X\right\|}_{2}^{2}+{\left\|Y\right\|}_{2}^{2}}=\frac{2\sum_{i=1}^{n}x_{i}y_{i}}{\sum_{i=1}^{n}x_{i}^{2}+\sum_{i=1}^{n}y_{i}^{2}}$$ (1)

$$J\left(X,Y\right)=\frac{X•Y}{{\left\|X\right\|}_{2}^{2}+{\left\|Y\right\|}_{2}^{2}-X•Y}=\frac{\sum_{i=1}^{n}x_{i}y_{i}}{\sum_{i=1}^{n}x_{i}^{2}+\sum_{i=1}^{n}y_{i}^{2}-\sum_{i=1}^{n}x_{i}y_{i}}$$ (2)

### Hesitant fuzzy linguistic term set

**Definition 1.** [16] Let *x*_*i*_ ∈ *X*, *i* = 1,2,…,*N*, and *S* = {s_*t*_|*t* = −*τ*,…,−1,0,1,…,*τ*} be a linguistic term set. Then, a hesitant fuzzy linguistic term set (HFLTS), *H*_*s*_ in *X*, is denoted as follows:

$$H_{s}=\left\{\left\langle x_{i},h_{s}(x_{i})\right\rangle |x_{i}\in X\right\}$$ (3)

where *h*_*s*_(*x*_*i*_) can be indicated as $h_{s}(x_{i})=\left\{s_{\varphi_{l}}(x_{i})|s_{\varphi_{l}}(x_{i})\in S,l=1,2,\ldots ,L(x_{i})\right\}$ where *φ*_*l*_ ∈ {−*τ*,…,−1,0,1,…,*τ*} is the subscript of a linguistic term and *L*(*x*_*i*_) is the total number of linguistic terms in *h*_*s*_(*x*_*i*_).

The linguistic terms of an HFLTS, $h_{s}=\left\{s_{\varphi_{l}}|l=1,2,\ldots ,L\right\}$, might be unordered. For simplicity, we arrange the linguistic terms, $s_{\varphi_{l}}(l=1,2,\ldots ,L)$, in ascending or descending order. The ascending order rule is to arrange the linguistic term set from small to large subscripts, whereas the descending order is just the opposite.

Different HFLTSs always possess different numbers of linguistic terms. Zhu and Xu [29] recommend a method for increasing the shorter HFLTs until it has the same length as the longer one. The adding regulation mainly relies upon the risk preferences of decision makers by adding the maximum value, minimum value, and mean value, which correspond to optimism, pessimism, and neutral rules, respectively. Without loss of generality, we add the shorter terms according to neutral rules in this paper.

### Similarity measures for HFLTSs

Let *S* = {s_*t*_|*t* = −*τ*,…,−1,0,1,…,*τ*} be a linguistic term set. For two HFLTSs, $H_{s}^{1}=\left\{\left\langle x_{i},h_{s}^{1}\left(x_{i}\right)\right\rangle |x_{i}\in X\right\}$ and $H_{s}^{2}=\left\{\left\langle x_{i},h_{s}^{2}\left(x_{i}\right)\right\rangle |x_{i}\in X\right\}$, Liao et al. [16] defined the information energy of $H_{s}^{1}$ and the correlation between two $H_{S}^{1}$ and $H_{s}^{2}$ as

$$E\left(H_{s}^{1}\right)=\sum_{i=1}^{N}\left(\frac{1}{L_{i}}\sum_{l=1}^{L_{i}}{\left(\frac{\delta_{l}^{1}\left(x_{i}\right)}{2\tau +1}\right)}^{2}\right)$$ (4)

$$C\left(H_{s}^{1},H_{s}^{2}\right)=\sum_{i=1}^{N}\left(\frac{1}{L_{i}}\sum_{l=1}^{L_{i}}\left(\frac{\left|\delta_{l}^{1}\left(x_{i}\right)\right|}{2\tau +1}\cdot \frac{\left|\delta_{l}^{2}\left(x_{i}\right)\right|}{2\tau +1}\right)\right)$$ (5),

respectively. Here, *L*_*i*_ is the maximum number of linguistic terms in $h_{s}^{1}(x_{i})$ or $h_{s}^{2}(x_{i})$ (with the shorter of the two needing to be extended to same length), $h_{s}^{k}(x_{i})=\left\{s_{\delta_{1}^{k}}(x_{i})|s_{\delta_{1}^{k}}(x_{i})\in S,l=1,\ldots ,L_{i}\right\},k=1,2,\cdots ,N$. The *N* is the cardinality of *X*. Based on the definitions of the information energy and correlation of the HFLTSs, two vector similarity measures for HFLTSs are proposed.

**Definition 2.** The similarity measure $D_{HFLTSs}(H_{s}^{1},H_{s}^{2})$ between $H_{S}^{1}$ and $H_{s}^{2}$ is defined as

$$D_{HFLTSs}(H_{s}^{1},H_{s}^{2})=\frac{2C\left(H_{s}^{1},H_{s}^{2}\right)}{{}^{{\left(E\left(H_{s}^{1}\right)\right)}^{}+{\left(E\left(H_{s}^{2}\right)\right)}^{}}}=\frac{2\sum_{i=1}^{N}\left(\frac{1}{L_{i}}\sum_{l=1}^{L_{i}}\left(\frac{\left|\delta_{l}^{1}\left(x_{i}\right)\right|}{2\tau +1}\cdot \frac{\left|\delta_{l}^{2}\left(x_{i}\right)\right|}{2\tau +1}\right)\right)}{\sum_{i=1}^{N}\left(\frac{1}{L_{i}}\sum_{l=1}^{L_{i}}{\left(\frac{\delta_{l}^{1}\left(x_{i}\right)}{2\tau +1}\right)}^{2}\right)+\sum_{i=1}^{N}\left(\frac{1}{L_{i}}\sum_{l=1}^{L_{i}}{\left(\frac{\delta_{l}^{2}\left(x_{i}\right)}{2\tau +1}\right)}^{2}\right)}.$$ (6)

Some theorems of similarity measure $D_{HFLTSs}(H_{s}^{1},H_{s}^{2})$ are proposed as follows:

**Theorem 1.** The similarity measure, $D_{HFLTSs}(H_{s}^{1},H_{s}^{2})$, between the HFLTSs, $H_{S}^{1}$ and $H_{s}^{2}$, possesses the following properties:

(1) $D_{HFLTSs}(H_{s}^{1},H_{s}^{2})=D_{HFLTSs}(H_{s}^{2},H_{s}^{1})$;
(2) $D_{HFLTSs}(H_{s}^{1},H_{s}^{2})=1$, if and only if $H_{s}^{1}=H_{s}^{2}$;
(3) $0\leq D_{HFLTSs}(H_{s}^{1},H_{s}^{2})\leq 1$.

**Proof**:

(1) and (2) are obvious.

(3) It is obvious for $D_{HFLTSs}(H_{s}^{1},H_{s}^{2})\geq 0$. According to the inequality *a*^2^ + *b*^2^ ≥ 2*ab*, we have $\sum_{i=1}^{N}\left(\frac{1}{L_{i}}\sum_{l=1}^{L_{i}}{\left(\frac{\delta_{l}^{1}\left(x_{i}\right)}{2\tau +1}\right)}^{2}\right)+\sum_{i=1}^{N}\left(\frac{1}{L_{i}}\sum_{l=1}^{L_{i}}{\left(\frac{\delta_{l}^{2}\left(x_{i}\right)}{2\tau +1}\right)}^{2}\right)\geq 2\sum_{i=1}^{N}\left(\frac{1}{L_{i}}\sum_{l=1}^{L_{i}}\left(\frac{\left|\delta_{l}^{1}\left(x_{i}\right)\right|}{2\tau +1}\cdot \frac{\left|\delta_{l}^{2}\left(x_{i}\right)\right|}{2\tau +1}\right)\right)$. Thus, we obtain $0\leq D_{HFLTSs}(H_{s}^{1},H_{s}^{2})\leq 1$, and the property (3) holds. □

**Definition 3.** Similarity measure $J_{HFLTSs}\left(H_{s}^{1},H_{s}^{2}\right)$ between $H_{S}^{1}$ and $H_{s}^{2}$ is defined as

$$\begin{array}{l} J_{HFLTSs}(H_{s}^{1},H_{s}^{2})=\frac{C\left(H_{s}^{1},H_{s}^{2}\right)}{{}^{E\left(H_{s}^{1}\right)+E\left(H_{s}^{2}\right)\text{-}C\left(H_{s}^{1},H_{s}^{2}\right)}}\\ =\frac{\sum_{i=1}^{N}\left(\frac{1}{L_{i}}\sum_{l=1}^{L_{i}}\left(\frac{\left|\delta_{l}^{1}\left(x_{i}\right)\right|}{2\tau +1}\cdot \frac{\left|\delta_{l}^{2}\left(x_{i}\right)\right|}{2\tau +1}\right)\right)}{\sum_{i=1}^{N}\left(\frac{1}{L_{i}}\sum_{l=1}^{L_{i}}{\left(\frac{\delta_{l}^{1}\left(x_{i}\right)}{2\tau +1}\right)}^{2}\right)+\sum_{i=1}^{N}\left(\frac{1}{L_{i}}\sum_{l=1}^{L_{i}}{\left(\frac{\delta_{l}^{2}\left(x_{i}\right)}{2\tau +1}\right)}^{2}\right)\text{-}\sum_{i=1}^{N}\left(\frac{1}{L_{i}}\sum_{l=1}^{L_{i}}\left(\frac{\left|\delta_{l}^{1}\left(x_{i}\right)\right|}{2\tau +1}\cdot \frac{\left|\delta_{l}^{2}\left(x_{i}\right)\right|}{2\tau +1}\right)\right)} \end{array}.$$ (7)

**Theorem 2.** The similarity measure, $J_{HFLTSs}\left(H_{s}^{1},H_{s}^{2}\right)$, between the HFLTSs $H_{S}^{1}$ and $H_{s}^{2}$ possesses the following properties:

(1) $J_{HFLTSs}(H_{s}^{1},H_{s}^{2})=J_{HFLTSs}(H_{s}^{2},H_{s}^{1})$;
(2) $J_{HFLTSs}(H_{s}^{1},H_{s}^{2})=1$, if and only if $H_{s}^{1}=H_{s}^{2}$;
(3) $0\leq J_{HFLTSs}(H_{s}^{1},H_{s}^{2})\leq 1$.

**Proof**:

(1) and (2) are obvious.

(3) This is obvious for $J_{HFLTSs}(H_{s}^{1},H_{s}^{2})\geq 0$. According to the inequality *a*^2^ + *b*^2^ ≥ 2*ab*, we have $\sum_{i=1}^{N}\left(\frac{1}{L_{i}}\sum_{l=1}^{L_{i}}{\left(\frac{\delta_{l}^{1}\left(x_{i}\right)}{2\tau +1}\right)}^{2}\right)+\sum_{i=1}^{N}\left(\frac{1}{L_{i}}\sum_{l=1}^{L_{i}}{\left(\frac{\delta_{l}^{2}\left(x_{i}\right)}{2\tau +1}\right)}^{2}\right)\text{-}\sum_{i=1}^{N}\left(\frac{1}{L_{i}}\sum_{l=1}^{L_{i}}\left(\frac{\left|\delta_{l}^{1}\left(x_{i}\right)\right|}{2\tau +1}\cdot \frac{\left|\delta_{l}^{2}\left(x_{i}\right)\right|}{2\tau +1}\right)\right)\geq \sum_{i=1}^{N}\left(\frac{1}{L_{i}}\sum_{l=1}^{L_{i}}\left(\frac{\left|\delta_{l}^{1}\left(x_{i}\right)\right|}{2\tau +1}\cdot \frac{\left|\delta_{l}^{2}\left(x_{i}\right)\right|}{2\tau +1}\right)\right)$. Thus, we obtain $0\leq J_{HFLTSs}(H_{s}^{1},H_{s}^{2})\leq 1$, and the property (3) holds. □

**Example 1.** Let *S* = {s_*α*_|*α* = −3,…,−1,0,1,…,3} be a linguistic term set and $H_{s}^{1}=\left\{s_{1},s_{2}\right\}$ and $H_{s}^{2}=\left\{s_{-3},s_{-1},s_{3}\right\}$ be two HFLTSs on *S*. We can extend $H_{s}^{1}$ to $H_{s}^{1}=\left\{s_{1},s_{1.5},s_{2}\right\}$ by adding the linguistic term, *s*_1.5_. Thus, the similarity measure, $J_{HFLTSs}\left(H_{s}^{1},H_{s}^{2}\right)$, between $H_{S}^{1}$ and $H_{s}^{2}$ is obtained as follows:

J=13(17×|−3|7+1.57×|−1|7+27×37)13((17)2+(1.57)2+(27)2)+13((−37)2+(−17)2+(37)2)−13(17×|−3|7+1.57×|−1|7+27×37)=10.5(3×49)15.75(3×49)=0.6667.

### Weighted similarity measures for HFLTSs

Let *w*_*i*_ be the weights of elements *x*_*i*_(i = 1,2,…,N) and $\sum_{i=1}^{N}w_{i}=1$. Then, the similarity measure formulas given in Eqs (6) and (7) can be extended as follows:

$$D^{'}{}_{HFLTSs}(H_{s}^{1},H_{s}^{2})=\frac{2C_{W}\left(H_{s}^{1},H_{s}^{2}\right)}{{}^{E_{W}\left(H_{s}^{1}\right)+E_{W}\left(H_{s}^{2}\right)}}=\frac{2\sum_{i=1}^{N}\left(\frac{w_{i}}{L_{i}}\sum_{l=1}^{L_{i}}\left(\frac{\left|\delta_{l}^{1}\left(x_{i}\right)\right|}{2\tau +1}\cdot \frac{\left|\delta_{l}^{2}\left(x_{i}\right)\right|}{2\tau +1}\right)\right)}{\sum_{i=1}^{N}\left(\frac{w_{i}}{L_{i}}\sum_{l=1}^{L_{i}}{\left(\frac{\delta_{l}^{1}\left(x_{i}\right)}{2\tau +1}\right)}^{2}\right)+\sum_{i=1}^{N}\left(\frac{w_{i}}{L_{i}}\sum_{l=1}^{L_{i}}{\left(\frac{\delta_{l}^{2}\left(x_{i}\right)}{2\tau +1}\right)}^{2}\right)}$$ (8)

$$\begin{array}{l} J^{'}{}_{HFLTSs}(H_{s}^{1},H_{s}^{2})=\frac{C_{w}\left(H_{s}^{1},H_{s}^{2}\right)}{{}^{E_{W}\left(H_{s}^{1}\right)+E_{W}\left(H_{s}^{2}\right)\text{-}C_{w}\left(H_{s}^{1},H_{s}^{2}\right)}}\\ =\frac{\sum_{i=1}^{N}\left(\frac{w_{i}}{L_{i}}\sum_{l=1}^{L_{i}}\left(\frac{\left|\delta_{l}^{1}\left(x_{i}\right)\right|}{2\tau +1}\cdot \frac{\left|\delta_{l}^{2}\left(x_{i}\right)\right|}{2\tau +1}\right)\right)}{\sum_{i=1}^{N}\left(\frac{w_{i}}{L_{i}}\sum_{l=1}^{L_{i}}{\left(\frac{\delta_{l}^{1}\left(x_{i}\right)}{2\tau +1}\right)}^{2}\right)+\sum_{i=1}^{N}\left(\frac{w_{i}}{L_{i}}\sum_{l=1}^{L_{i}}{\left(\frac{\delta_{l}^{2}\left(x_{i}\right)}{2\tau +1}\right)}^{2}\right)\text{-}\sum_{i=1}^{N}\left(\frac{w_{i}}{L_{i}}\sum_{l=1}^{L_{i}}\left(\frac{\left|\delta_{l}^{1}\left(x_{i}\right)\right|}{2\tau +1}\cdot \frac{\left|\delta_{l}^{2}\left(x_{i}\right)\right|}{2\tau +1}\right)\right)} \end{array}$$ (9)

It is obvious that, if w=(1n,1n,⋯,1n), then Eqs (8) and (9) are simplified to Eqs (6) and (7), respectively. Likewise, the two weighted similarity measures also possess the following properties:

(1) $D^{'}{}_{HFLTSs}(H_{s}^{1},H_{s}^{2})=D^{'}{}_{HFLTSs}(H_{s}^{2},H_{s}^{1}),$ $J^{'}{}_{HFLTSs}(H_{s}^{1},H_{s}^{2})=J^{'}{}_{HFLTSs}(H_{s}^{2},H_{s}^{1})$;
(2) $0\leq D^{'}{}_{HFLTSs}(H_{s}^{1},H_{s}^{2}),J^{'}{}_{HFLTSs}(H_{s}^{1},H_{s}^{2})\leq 1$;
(3) $D^{'}{}_{HFLTSs}(H_{s}^{1},H_{s}^{2})=J^{'}{}_{HFLTSs}(H_{s}^{1},H_{s}^{2})=1$, if and only if $H_{s}^{1}=H_{s}^{2}$.

### Ordered weighted similarity measure for HFLTSs

Inspired by the OWA operators proposed by Yager [30], Liao et al. [17] defined the ordered weighted correlation of any two HFLTSs, $H_{S}^{1}$ and $H_{s}^{2}$, by

$$C_{ow}(H_{s}^{1},H_{s}^{2})=\sum_{i=1}^{N}\left(\frac{w_{i}}{L_{\zeta (i)}}\sum_{l=1}^{L_{\zeta (i)}}\left(\frac{\left|\delta_{l}^{1}\left(x_{\zeta (i)}\right)\right|}{2\tau +1}\cdot \frac{\left|\delta_{l}^{2}\left(x_{\zeta (i)}\right)\right|}{2\tau +1}\right)\right)$$ (10)

Where *ζ*(1),*ζ*(2),…,*ζ*(N) satisfy

$$\frac{1}{L_{\zeta (i)}}\sum_{l=1}^{L_{\zeta (i)}}\left(\frac{\left|\delta_{l}^{1}\left(x_{\zeta (i)}\right)\right|}{2\tau +1}\cdot \frac{\left|\delta_{l}^{2}\left(x_{\zeta (i)}\right)\right|}{2\tau +1}\right)\geq \frac{1}{L_{\zeta (i+1)}}\sum_{l=1}^{L_{\zeta (i+1)}}\left(\frac{\left|\delta_{l}^{1}\left(x_{\zeta (i+1)}\right)\right|}{2\tau +1}\cdot \frac{\left|\delta_{l}^{2}\left(x_{\zeta (i+1)}\right)\right|}{2\tau +1}\right)$$ (11)

Here, *w*_*i*_ are the weights of ordered positions for elements *x*_*i*_(i = 1,2,…,N) with $\sum_{i=1}^{N}w_{i}=1$.

Similarly, the ordered weighed information energy of the set, *H*_*s*_, is defined as

$$E_{ow}(H_{s})=\sum_{i=1}^{N}\left(\frac{w_{i}}{L_{\zeta (i)}}\sum_{l=1}^{L_{\zeta (i)}}{\left(\frac{\delta_{l}^{}\left(x_{\zeta (i)}\right)}{2\tau +1}\right)}^{2}\right)$$ (12)

Afterwards, we extend Eqs (8) and (9) to Eqs (13) and (14), respectively:

$$D^{''}{}_{HFLTSs}(H_{s}^{1},H_{s}^{2})=\frac{2C_{oW}\left(H_{s}^{1},H_{s}^{2}\right)}{{}^{E_{oW}\left(H_{s}^{1}\right)+E_{oW}\left(H_{s}^{2}\right)}}=\frac{2\sum_{i=1}^{N}\left(\frac{w_{i}}{L_{\zeta (i)}}\sum_{l=1}^{L_{\zeta (i)}}\left(\frac{\left|\delta_{l}^{1}\left(x_{\zeta (i)}\right)\right|}{2\tau +1}\cdot \frac{\left|\delta_{l}^{2}\left(x_{\zeta (i)}\right)\right|}{2\tau +1}\right)\right)}{\sum_{i=1}^{N}\left(\frac{w_{i}}{L_{\zeta (i)}}\sum_{l=1}^{L_{\zeta (i)}}{\left(\frac{\delta_{l}^{1}\left(x_{\zeta (i)}\right)}{2\tau +1}\right)}^{2}\right)+\sum_{i=1}^{N}\left(\frac{w_{i}}{L_{\zeta (i)}}\sum_{l=1}^{L_{\zeta (i)}}{\left(\frac{\delta_{l}^{2}\left(x_{\zeta (i)}\right)}{2\tau +1}\right)}^{2}\right)}$$ (13)

$$\begin{array}{l} J^{''}{}_{HFLTSs}(H_{s}^{1},H_{s}^{2})=\frac{C_{ow}\left(H_{s}^{1},H_{s}^{2}\right)}{{}^{E_{ow}\left(H_{s}^{1}\right)+E_{ow}\left(H_{s}^{2}\right)\text{-}C_{ow}\left(H_{s}^{1},H_{s}^{2}\right)}}\\ =\frac{\sum_{i=1}^{N}\left(\frac{w_{i}}{L_{\zeta (i)}}\sum_{l=1}^{L_{\zeta (i)}}\left(\frac{\left|\delta_{l}^{1}\left(x_{\zeta (i)}\right)\right|}{2\tau +1}\cdot \frac{\left|\delta_{l}^{2}\left(x_{\zeta (i)}\right)\right|}{2\tau +1}\right)\right)}{\sum_{i=1}^{N}\left(\frac{w_{i}}{L_{\zeta (i)}}\sum_{l=1}^{L_{\zeta (i)}}{\left(\frac{\delta_{l}^{1}\left(x_{\zeta (i)}\right)}{2\tau +1}\right)}^{2}\right)+\sum_{i=1}^{N}\left(\frac{w_{i}}{L_{\zeta (i)}}\sum_{l=1}^{L_{\zeta (i)}}{\left(\frac{\delta_{l}^{2}\left(x_{\zeta (i)}\right)}{2\tau +1}\right)}^{2}\right)-\sum_{i=1}^{N}\left(\frac{w_{i}}{L_{\zeta (i)}}\sum_{l=1}^{L_{\zeta (i)}}\left(\frac{\left|\delta_{l}^{1}\left(x_{\zeta (i)}\right)\right|}{2\tau +1}\cdot \frac{\left|\delta_{l}^{2}\left(x_{\zeta (i)}\right)\right|}{2\tau +1}\right)\right)} \end{array}$$ (14)

The two ordered, weighted similarity measures also have the following properties:

(1) $D^{''}{}_{HFLTSs}(H_{s}^{1},H_{s}^{2})=D^{''}{}_{HFLTSs}(H_{s}^{2},H_{s}^{1})$, $J^{''}{}_{HFLTSs}(H_{s}^{1},H_{s}^{2})=J^{''}{}_{HFLTSs}(H_{s}^{2},H_{s}^{1})$;
(2) $0\leq D^{''}{}_{HFLTSs}(H_{s}^{1},H_{s}^{2}),J^{''}{}_{HFLTSs}(H_{s}^{1},H_{s}^{2})$;
(3) $D^{''}{}_{HFLTSs}(H_{s}^{1},H_{s}^{2})=J^{''}{}_{HFLTSs}(H_{s}^{1},H_{s}^{2})=1$, if and only if $H_{s}^{1}=H_{s}^{2}$.

### The $D_{HFLTSs}(H_{s}^{1},H_{s}^{2})$-distance-based HFL-TOPSIS method

In recent years, multi-criteria decision making (MCDM) methods [31–36] have been developed and widely applied to diverse scientific fields, such as water resource utilization, energy management, machine tool evaluation, and supplier selection. TOPSIS is a simple and widely used MCDM method [37–39] for order preference using a close-to-ideal solution [40–42]. With the development of fuzzy sets, TOPSIS has been extended to fuzzy environments [43–48]. Furthermore, distance and similarity measures have a mutual transformation relationship with each other. Liao et al. [15] defined this relationship for HFLTSs as follows:

$$d\left(H_{s}^{1},H_{s}^{2}\right)=1-\rho \left(H_{s}^{1},H_{s}^{2}\right)$$ (15)

Then, the corresponding distance measures can be easily obtained using Eq (15). Inspired by the cosine-distance-based HFL-TOPSIS method [16], the $D_{HFLTSs}(H_{s}^{1},H_{s}^{2})$-distance-based HFL-TOPSIS method can be defined as follows:

**Step 1.** Let *A* = {*A*_1_,*A*_2_,⋯,*A*_*n*_} and *C* = {*C*_1_,*C*_2_,⋯,*C*_*m*_} be a set of alternatives and a set of criteria, respectively. Let *w*_*j*_ be the weights of criteria *C*_*j*_, where $0\leq \omega_{j}\leq 1\left(j=1,2,\ldots ,m\right),\sum_{j=1}^{m}\omega_{j}=1$. The characteristics of *A*_*i*_ in relation to criteria *c*_*j*_ are represented by an HFLE, $h_{s}^{ij}$, where *S* = {s_*t*_|*t* = −*τ*,…,−1,0,1,…,*τ*} is a linguistic term set.
**Step 2**. The positive ideal solution, $A^{+}=\left\{h_{s}^{1+},h_{s}^{2+},\ldots ,h_{s}^{m+}\right\}$, and negative ideal solution, $A^{-}=\left\{h_{s}^{1-},h_{s}^{2-},\ldots ,h_{s}^{m-}\right\}$, are developed as follows:
  $$h_{s}^{j+}=\{\begin{array}{c} \underset{i=1,2,\ldots ,n}{\max}h_{s}^{ij+},\;{\mathrm{benefit}\;\mathrm{criterion}\;C}_{j}\\ \underset{i=1,2,\ldots ,n}{\min}h_{s}^{ij+},\;{\mathrm{cost}\;\mathrm{criterion}\;C}_{j} \end{array},\;\mathrm{for}\;j=1,2,\ldots ,m$$
  $$h_{s}^{j-}=\{\begin{array}{c} \underset{i=1,2,\ldots ,n}{\min}h_{s}^{ij-},\;{\mathrm{benefit}\;\mathrm{criterion}\;C}_{j}\\ \underset{i=1,2,\ldots ,n}{\max}h_{s}^{ij-},\;{\mathrm{cost}\;\mathrm{criterion}\;C}_{j} \end{array},\;\mathrm{for}\;j=1,2,\ldots ,m$$
**Step 3.** According to Eq (16), the construction of the positive ideal distance matrix, D^+^, and the negative ideal distance matrix, D^−^, are given as
  $$D^{+}=\left[\begin{array}{cccc} d_{*}(h_{s}^{11},h_{s}^{1+}) & d_{*}(h_{s}^{12},h_{s}^{2+}) & \ldots & d_{*}(h_{s}^{1m},h_{s}^{m+})\\ d_{*}(h_{s}^{21},h_{s}^{1+}) & d_{*}(h_{s}^{22},h_{s}^{2+}) & \ldots & d_{*}(h_{s}^{2m},h_{s}^{m+})\\ \vdots & \vdots & \ddots & \vdots \\ d_{*}(h_{s}^{n1},h_{s}^{1+}) & d_{*}(h_{s}^{n2},h_{s}^{2+}) & \ldots & d_{*}(h_{s}^{nm},h_{s}^{m+}) \end{array}\right]$$
  $$D^{-}=\left[\begin{array}{cccc} d_{*}(h_{s}^{11},h_{s}^{1-}) & d_{*}(h_{s}^{12},h_{s}^{2-}) & \ldots & d_{*}(h_{s}^{1m},h_{s}^{m-})\\ d_{*}(h_{s}^{21},h_{s}^{1-}) & d_{*}(h_{s}^{22},h_{s}^{2-}) & \ldots & d_{*}(h_{s}^{2m},h_{s}^{m-})\\ \vdots & \vdots & \ddots & \vdots \\ d_{*}(h_{s}^{n1},h_{s}^{1-}) & d_{*}(h_{s}^{n2},h_{s}^{2-}) & \ldots & d_{*}(h_{s}^{nm},h_{s}^{m-}) \end{array}\right].$$
  where the distance between the two HFLEs, $h_{s}^{1}$ and $h_{s}^{2}$, can be given as follows:
  $$d_{*}\left(h_{s}^{1},h_{s}^{2}\right)=1-\frac{2\sum_{i=1}^{N}\left(\frac{1}{L_{i}}\sum_{l=1}^{L_{i}}\left(\frac{\left|\delta_{l}^{1}\left(x_{i}\right)\right|}{2\tau +1}\cdot \frac{\left|\delta_{l}^{2}\left(x_{i}\right)\right|}{2\tau +1}\right)\right)}{\sum_{i=1}^{N}\left(\frac{1}{L_{i}}\sum_{l=1}^{L_{i}}{\left(\frac{\delta_{l}^{1}\left(x_{i}\right)}{2\tau +1}\right)}^{2}\right)+\sum_{i=1}^{N}\left(\frac{1}{L_{i}}\sum_{l=1}^{L_{i}}{\left(\frac{\delta_{l}^{2}\left(x_{i}\right)}{2\tau +1}\right)}^{2}\right)}$$ (16)
**Step 4.** Calculate the closeness coefficient
  $$R_{i}=\frac{D_{i}^{-}}{D_{i}^{-}+D_{i}^{+}}$$ (17)
  Where $D_{i}^{+}=\sum_{j=1}^{m}\omega_{j}d_{*}(h_{s}^{ij},h_{s}^{j+})$ and $D_{i}^{-}=\sum_{j=1}^{m}\omega_{j}d_{*}(h_{s}^{ij},h_{s}^{j-})$.
**Step 5.** Rank the alternatives by decreasing order of *R*_*i*_.

In the same way, the distance with the $J_{HFLTSs}\left(H_{s}^{1},H_{s}^{2}\right)$-distance-based HFL-TOPSIS method can be denoted as follows:

$$d_{*}\left(h_{s}^{1},h_{s}^{2}\right)=1-\frac{\sum_{i=1}^{N}\left(\frac{1}{L_{i}}\sum_{l=1}^{L_{i}}\left(\frac{\left|\delta_{l}^{1}\left(x_{i}\right)\right|}{2\tau +1}\cdot \frac{\left|\delta_{l}^{2}\left(x_{i}\right)\right|}{2\tau +1}\right)\right)}{\sum_{i=1}^{N}\left(\frac{1}{L_{i}}\sum_{l=1}^{L_{i}}{\left(\frac{\delta_{l}^{1}\left(x_{i}\right)}{2\tau +1}\right)}^{2}\right)+\sum_{i=1}^{N}\left(\frac{1}{L_{i}}\sum_{l=1}^{L_{i}}{\left(\frac{\delta_{l}^{2}\left(x_{i}\right)}{2\tau +1}\right)}^{2}\right)\text{-}\sum_{i=1}^{N}\left(\frac{1}{L_{i}}\sum_{l=1}^{L_{i}}\left(\frac{\left|\delta_{l}^{1}\left(x_{i}\right)\right|}{2\tau +1}\cdot \frac{\left|\delta_{l}^{2}\left(x_{i}\right)\right|}{2\tau +1}\right)\right)}$$ (18)

## Application of the similarity measures of HFLTS

**Example 2** [17]. In traditional Chinese medical diagnosis, a doctor always gets some imprecise information about a patient’s symptoms, such as temperature, headache, cough, and stomach pain, through seeing, smelling, asking, and touching. Assuming that a doctor wants to make a proper diagnosis for a patient with four symptoms, V = {temperature, headache, cough, stomach pain} with four possible diseases, D = {Viral infection, Typhoid, Pneumonia, Stomach problem}. Each symptom can be seen as a linguistic variable, whose corresponding linguistic term set is shown as follows:

$$S_{1}=\left\{s_{-3}=very\;{\mathrm{low},\;s}_{-2}=low,{\;s}_{-1}=a\;little\;{\mathrm{low},\;s}_{0}=medium,{\;s}_{1}=a\;little\;{\mathrm{high},\;s}_{2}=high,\;s_{3}=very\;\mathrm{high}\right\},$$

$$S_{2}=\left\{s_{-3}=none{,s}_{-2}=very\;\mathrm{slight},s_{-1}=slight{,s}_{0}=a\;\mathrm{little}\;\mathrm{terrible},s_{1}=terrible{,s}_{2}=very\;terrible,s_{3}=insufferable\right\},$$

$$S_{3}=\left\{s_{-3}=none{,s}_{-2}=very\;\mathrm{slight},s_{-1}=slight{,s}_{0}=a\;\mathrm{little}\;\mathrm{serious},s_{1}={\mathrm{serious},s}_{2}=very\;\mathrm{serious},s_{3}=insufferable\right\},$$

$$S_{4}=\left\{s_{-3}=none{,s}_{-2}=very\;\mathrm{slight},s_{-1}=slight{,s}_{0}=a\;\mathrm{little}\;\mathrm{terrible},s_{1}={\mathrm{terrible},s}_{2}=very\;\mathrm{terrible},s_{3}=insufferable\right\}.$$

We can generate the following knowledge-based data set in terms of HFLTSs (see Table 1) according to existing experience. Assume there are four patients, P = {Richard, Catherine, Nicole, Kevin}, whose symptoms, as linguistic expressions, can be transformed into HFLTSs (Table 2).

**Table 1:** Symptoms characteristic for the considered diagnosis in terms of HFLTSs.

	Temperature	Headache	Cough	Stomach pain
Viral infection	{s_1_,*s*_2_,*s*_3_}	{*s*_0_,*s*_1_,*s*_2_}	{s_1_,*s*_2_,*s*_3_}	{*s*_−3_}
Typhoid	{*s*_2_,*s*_3_}	{s_1_,*s*_2_,*s*_3_}	{s_1_,*s*_2_,*s*_3_}	{*s*_-3_,*s*_-2_}
Pneumonia	{*s*_0_,*s*_1_}	{*s*_−1_,*s*_0_}	{*s*_2_,*s*_3_}	{*s*_−3_}
Stomach problem	{*s*_0_}	{*s*_−3_}	{*s*_−3_}	{s_1_,*s*_2_,*s*_3_}

**Table 2:** Symptoms characteristic for the considered patients in terms of HFLTSs.

	Temperature	Headache	Cough	Stomach pain
Richard	{*s*_2_}	{*s*_2_}	{s_1_,*s*_2_}	{*s*_−3_}
Catherine	{*s*_0_}	{*s*_−3_}	{*s*_−3_}	{s_1_,*s*_2_}
Nicole	{*s*_3_}	{*s*_1_}	{*s*_2_}	{*s*_−3_}
Kevin	{*s*_1_}	{s_−1_,*s*_0_}	{*s*_2_}	{*s*_−3_}

In order to diagnosis the diseases of these four patients, we can calculate the similarity measures between the data set of each patient’s symptoms and that of the diagnoses. We use two new similarity measures, $D_{HFLTSs}\left(H_{s}^{1},H_{s}^{2}\right)$ and $J_{HFLTSs}\left(H_{s}^{1},H_{s}^{2}\right)$, to derive the relationship between each patient and disease, and the similarity values taken from the Dice and Jaccard measures are displayed in Tables 3 and 4, respectively.

**Table 3:** Similarity values of $D_{HFLTSs}\left(H_{s}^{1},H_{s}^{2}\right)$ between each patient’s symptoms and possible diagnosis.

	Viral infection	Typhoid	Pneumonia	Stomach problem
Richard	0.9283	**0.9482**	0.8333	0.7826
Catherine	0.6667	0.7237	0.7297	**0.9884**
Nicole	**0.9302**	0.9265	0.8101	0.6569
Kevin	0.8792	0.7964	**0.9677**	0.7265

**Table 4:** Similarity values of $J_{HFLTSs}\left(H_{s}^{1},H_{s}^{2}\right)$ between each patient’s symptoms and possible diagnosis.

	Viral infection	Typhoid	Pneumonia	Stomach problem
Richard	0.8661	**0.9015**	0.7143	0.6428
Catherine	0.5000	0.5671	0.5745	**0.9771**
Nicole	**0.8696**	0.8630	0.6809	0.4891
Kevin	0.7845	0.6617	**0.9375**	0.5704

The principle behind the diagnosis is the larger the value of the similarity measure, the higher possibility of the diagnosis for the patient. From Table 3 and Table 4, we can see that Richard, Catherine, Nicole, and Kevin are suffering from typhoid, stomach problem, viral fever, and pneumonia, respectively, which is in concordance with the correlation coefficient values of *ρ*_1_ calculated in [17], but not with those of *ρ*_2_ calculated in [17]. This is because the $D_{HFLTSs}(H_{s}^{1},H_{s}^{2})$ and $J_{HFLTSs}\left(H_{s}^{1},H_{s}^{2}\right)$ similarity measures are obtained according to the normalized inner product within a vector space, while *ρ*_2_ [17] was defined using the classical overlap measure. These two different measures come from different points of view.

**Example 3.** In the following, we discuss an MCDM problem [16] in terms of both the $D_{HFLTSs}(H_{s}^{1},H_{s}^{2})$- and $J_{HFLTSs}\left(H_{s}^{1},H_{s}^{2}\right)$-distance-based HFL-TOPSIS methods, respectively.

Assume a company intends to select an ERP system from three candidates, *A* = {*A*_1_,*A*_2_,*A*_3_}, with three criteria: *C*_1_ (potential cost), *C*_2_ (function), and *C*_3_ (operational complexity) of weights 0.3, 0.5, and 0.2, respectively. As ERP systems are very complicated, it is not easy to use just one linguistic term to express an opinion for the decision maker. Thus, the decision maker may be hesitant when determining the values of each ERP system over the criteria. We transform the linguistic expressions of CIO (Chief Information Officer) into a HFLTS judgment matrix *H*, using the transformation function [25]:

$$H=\left[\begin{array}{ccc} \left\{s_{1},s_{2},s_{3}\right\} & \left\{s_{2},s_{3}\right\} & \left\{s_{1},s_{2},s_{3}\right\}\\ \left\{s_{1},s_{2},s_{3}\right\} & \left\{s_{1},s_{2},s_{3}\right\} & \left\{s_{-2},s_{-1},s_{0}\right\}\\ \left\{s_{2},s_{3}\right\} & \left\{s_{1},s_{2},s_{3}\right\} & \left\{s_{3}\right\} \end{array}\right].$$

Now, we try to use the $D_{HFLTSs}(H_{s}^{1},H_{s}^{2})$- and $J_{HFLTSs}\left(H_{s}^{1},H_{s}^{2}\right)$-distance-based HFL-TOPSIS methods to solve this MCDM problem.

(1) Using the $D_{HFLTSs}(H_{s}^{1},H_{s}^{2})$-distance-based HFL-TOPSIS method

**Step 1.** It is given above, so we go to Step 2 directly;
**Step 2.** Since all criteria are benefit criteria according to the score function and the variance function [49], we obtain $MAX(C_{1})=h_{s}^{31}=\left\{s_{2},s_{3}\right\}$, $\mathrm{MIN}(C_{1})=h_{s}^{11}=h_{s}^{21}=\left\{s_{1},s_{2},s_{3}\right\}$, $MAX(C_{2})=h_{s}^{12}=\left\{s_{2},s_{3}\right\}$, $\mathrm{MIN}(C_{2})=h_{s}^{22}=h_{s}^{32}=\left\{s_{1},s_{2},s_{3}\right\}$, $MAX(C_{3})=h_{s}^{33}=\left\{s_{3}\right\}$, and $\mathrm{MIN}(C_{3})=h_{s}^{23}=\left\{s_{\text{-}2},s_{\text{-}1},s_{0}\right\}$. Thus, the positive ideal solution and the negative ideal solution for this problem are *A*^+^ = ({*s*_2_,*s*_3_},{*s*_2_,*s*_3_},{*s*_3_})^*T*^ and *A*^−^ = ({*s*_1_,*s*_2_,*s*_3_},{*s*_1_,*s*_2_,*s*_3_},{*s*_−2_,*s*_−1_,*s*_0_})^*T*^, respectively.
**Step 3.** According to Eq (16), we can construct *D*^+^ and *D*^−^ as follows:
  $$D^{+}=\left[\begin{array}{ccc} 0.0376 & 0 & 0.1220\\ 0.0376 & 0.0376 & 0.4375\\ 0 & 0.0376 & 0 \end{array}\right],\;D^{-}=\left[\begin{array}{ccc} 0 & 0.0376 & 0.5789\\ 0 & 0 & 0\\ 0.0376 & 0 & 0.4375 \end{array}\right].$$
**Step 4.** Using Eq (17), we can calculate the closeness coefficient. Since $D_{1}^{+}=0.03568$, $D_{2}^{+}=0.11758$, $D_{3}^{+}=0.0188$, $D_{1}^{-}=0.13458$, $D_{2}^{-}=0$, $D_{3}^{-}=0.09878$, we obtain *RC*(*A*_1_) = 0.7904, *RC*(*A*_2_) = 0, *RC*(*A*_3_) = 0.8401.
**Step 5.** By means of the closeness coefficient of each alternative, the ranking of these ERP systems is *A*_3_ ≻ *A*_1_ ≻ *A*_2_, which implies that the third ERP system, *A*_3_, is the best choice for the company.

(2) Using the $J_{HFLTSs}\left(H_{s}^{1},H_{s}^{2}\right)$-distance-based HFL-TOPSIS method

**Steps 1 and 2** are the same as those in the -distance-based HFL-TOPSIS method;
**Step 3.** According to Eq (18), we can construct *D*^+^ and *D*^−^ as follows:
  $$D^{+}=\left[\begin{array}{ccc} 0.0725 & 0 & 0.2174\\ 0.0725 & 0.0725 & 0.6087\\ 0 & 0.0725 & 0 \end{array}\right],\;D^{-}=\left[\begin{array}{ccc} 0 & 0.0725 & 0.7333\\ 0 & 0 & 0\\ 0.0725 & 0 & 0.6087 \end{array}\right].$$
**Step 4.** Using Eq (17), calculate the closeness coefficient. Since $D_{1}^{+}=0.06523$, $D_{2}^{+}=0.17974$, $D_{3}^{+}=0.03625$, $D_{1}^{-}=0.18291$, $D_{2}^{-}=0$, $D_{3}^{-}=0.14349$, we obtain *RC*(*A*_1_) = 0.73712, *RC*(*A*_2_) = 0, *RC*(*A*_3_) = 0.79832.
**Step 5.** By means of the closeness coefficient of each alternative, the ranking of these ERP systems is *A*_3_ ≻ *A*_1_ ≻ *A*_2_, which implies that the third ERP system, *A*_3_, is the best choice for the company.

From the above results, it can be concluded that the ranking of our two TOPSIS methods is the same, while that [16] of the three ERP systems is inconsistent as a result of the application of different distance measures to TOPSIS methods. However, the closeness coefficients of the third and first ERP systems are very similar to that of the ideal solution. In fact, the third ERP system, *A*_3_ = ({*s*_2_,*s*_3_},{*s*_1_,*s*_2_,*s*_3_},{*s*_3_})^*T*^, is closer to the positive ideal solution, *A*^+^ = ({*s*_2_,*s*_3_},{*s*_2_,*s*_3_},{*s*_3_})^*T*^, than the first ERP system *A*_1_ = ({*s*_1_,*s*_2_,*s*_3_},{*s*_2_,*s*_3_},{*s*_1_,*s*_2_,*s*_3_})^*T*^. Therefore, the third ERP system is a better choice for the company than the first ERP system, which validates that our methods are effective. The ranking result should be regarded as a support to the decision-making process. Afterwards, decision makers can choose an ERP system according to their preferences based on the ranking results of the TOPSIS method.

**Example 4.** Suppose that assessed values of two alternatives are *A*_1_ = ({*s*_2_,*s*_1_,*s*_−1_},{*s*_2_,*s*_1_},{*s*_2_,*s*_1_,*s*_−1_}), *A*_2_ = ({*s*_3_,*s*_2_,*s*_1_},{*s*_2_,*s*_1_},{*s*_−1_,*s*_−2_}) based on three criteria weight vector *ω* = (0.35,0.25,0.4), and ideal alternative is A^*^ = ({*s*_4_,*s*_4_,*s*_4_},{*s*_4_,*s*_4_},{*s*_4_,*s*_4_}).

In the following, we calculate the Dice, Jaccard, and cosine [16] similarity measures between *A*_1_ and A^*^, and Dice, Jaccard, and cosine similarity measures between *A*_2_ and A^*^, respectively.

$$D_{HFLTSs}\left(A_{1},A^{*}\right)=0.35\times 0.594+0.25\times 0.641+0.4\times 0.595=0.6062$$

$$D_{HFLTSs}\left(A_{2},A^{*}\right)=0.35\times 0.778+0.25\times 0.641+0.4\times 0.653=0.6938.$$

$$J_{HFLTSs}\left(A_{1},A^{*}\right)=0.35\times 0.423+0.25\times 0.4713+0.4\times 0.4239=0.4354.$$

$$J_{HFLTSs}\left(A_{2},A^{*}\right)=0.35\times 0.6362+0.25\times 0.4713+0.4\times 0.4843=0.5342.$$

$$C_{HFLTSs}\left(A_{1},A^{*}\right)=0.35\times 0.9447+0.25\times 0.941+0.4\times 0.947=0.9447.$$

$$C_{HFLTSs}\left(A_{2},A^{*}\right)=0.35\times 0.929+0.25\times 0.941+0.4\times 0.9634=0.9458.$$

Although above three similarities obtain same conclusion that *A*_2_ is better than *A*_1_, *C*_*HFLTSs*_(*A*_1_,A^*^) is approximately equal *C*_*HFLTSs*_(*A*_2_,A^*^). Therefore, it means that Dice and Jaccard similarities have a stronger ability to discriminate between HFLTSs than Cosine similarity, which could further verify our conclusions, namely, the Dice and Jaccard similarity measures are more reasonable than the cosine similarity measure with respect to HFLTSs.

## Conclusions

In this paper, we introduced two novel similarity measures for HFLTSs and enumerated some properties of these similarity measures. Furthermore, the two weighted similarity measures and the ordered weighted similarity measures for HFLTSs have been established and analyzed. Inspired by the cosine-distance-based HFL-TOPSIS method, the $D_{HFLTSs}(H_{s}^{1},H_{s}^{2})$- and $J_{HFLTSs}\left(H_{s}^{1},H_{s}^{2}\right)$-distance-based HFL-TOPSIS methods can be introduced. An application example concerning the traditional Chinese medical diagnosis and a MCDM problem have been discussed to illustrate the applicability and validation of both our HFLTS similarity measures. Through examples, it has been shown that the Dice and Jaccard measures are more reasonable than the cosine measure for the HFLTS.
